# The carbon–iodine bond cleavage and isomerization of iodoform visualized with femtosecond X-ray liquidography†

**DOI:** 10.1039/d4sc04604h

**Published:** 2024-10-29

**Authors:** Yongjun Cha, Hosung Ki, Donghwan Im, Yunbeom Lee, Seonggon Lee, Jungmin Kim, Jae Hyuk Lee, Jeongho Kim, Hyotcherl Ihee

**Affiliations:** a Department of Chemistry, Korea Advanced Institute of Science and Technology (KAIST) Daejeon 34141 Republic of Korea hyotcherl.ihee@kaist.ac.kr; b Center for Advanced Reaction Dynamics (CARD), Institute for Basic Science (IBS) Daejeon 34141 Republic of Korea; c Pohang Accelerator Laboratory Pohang 37673 Republic of Korea; d Department of Chemistry, Inha University 100 Inha-ro, Michuhol-gu Incheon 22212 Republic of Korea

## Abstract

Iodoform (CHI_3_) has garnered significant attention for its unique ability to induce photo-cyclopropanation of olefins by releasing an iodine radical through C–I bond cleavage. However, the detailed mechanism underlying CHI_3_ photodissociation is still not fully understood. Here, we elucidate the ultrafast structural dynamics of CHI_3_ upon photoexcitation using femtosecond time-resolved X-ray liquidography (fs-TRXL) at an X-ray free-electron laser facility. The fs-TRXL data was decomposed into the isotropic and anisotropic data. The isotropic data reveal that the formation of CHI_2_ and I radicals upon photolysis precedes the emergence of *iso*-CHI_2_–I. After a short induction period, two competing geminate recombination pathways of CHI_2_ and I radicals take place: one pathway leads to the recovery of CHI_3_, while the other results in the formation of *iso*-CHI_2_–I. Additionally, the anisotropic data show how the transient anisotropic distribution of both the species formed upon photoexcitation and the ground-state species depleted upon photoexcitation decays through rotational dephasing. Furthermore, the observed structural dynamics of CHI_3_ has distinctive differences with that of BiI_3_, which can be attributed to differences in their central moieties, CH and Bi. Our findings provide insights into the photoinduced reaction dynamics of CHI_3_, enhancing the understanding of its role in photochemical reactions.

## Abstract 2

The photolysis process of iodoform (CHI_3_), involving C–I bond cleavage, geminate recombination, and rotational dephasing, has been visualized using femtosecond X-ray liquidography.

## Graphical abstract

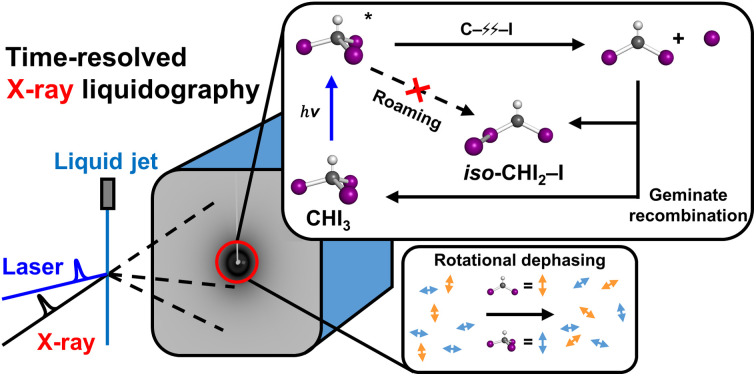

## Introduction

Haloalkanes have long captured the attention of researchers due to their diverse applications, finding utility in areas that demand halogen radicals or anions.^1^ Their significance stems from their innate tendency to undergo carbon–halogen bond cleavage through ultraviolet photolysis, making them effective halogen-releasing agents. Among the haloalkanes, polyhalomethanes have garnered extensive studies, primarily due to their unique ability to recombine with released halogen radicals, resulting in the formation of *iso*-polyhalomethanes.^2–6^

The generation of *iso*-polyhalomethanes can be categorized into two primary mechanisms: (1) solvent cage-mediated geminate recombination and (2) roaming reaction. Traditionally, *iso*-polyhalomethanes were believed to be exclusively formed *via* solvent cage-mediated geminate recombination. However, recent studies on CHBr_3_ ^7^ and BiI_3_,^8^ a polyhalomethane and a polyhalomethane-like system, respectively, have unveiled a novel isomerization process mediated by roaming reactions. In particular, the occurrence of roaming-mediated isomer formation in <200 fs time domain in the photolysis pathways of BiI_3_ was revealed *via* femtosecond time-resolved X-ray liquidography (fs-TRXL).

**Fig. 1:**
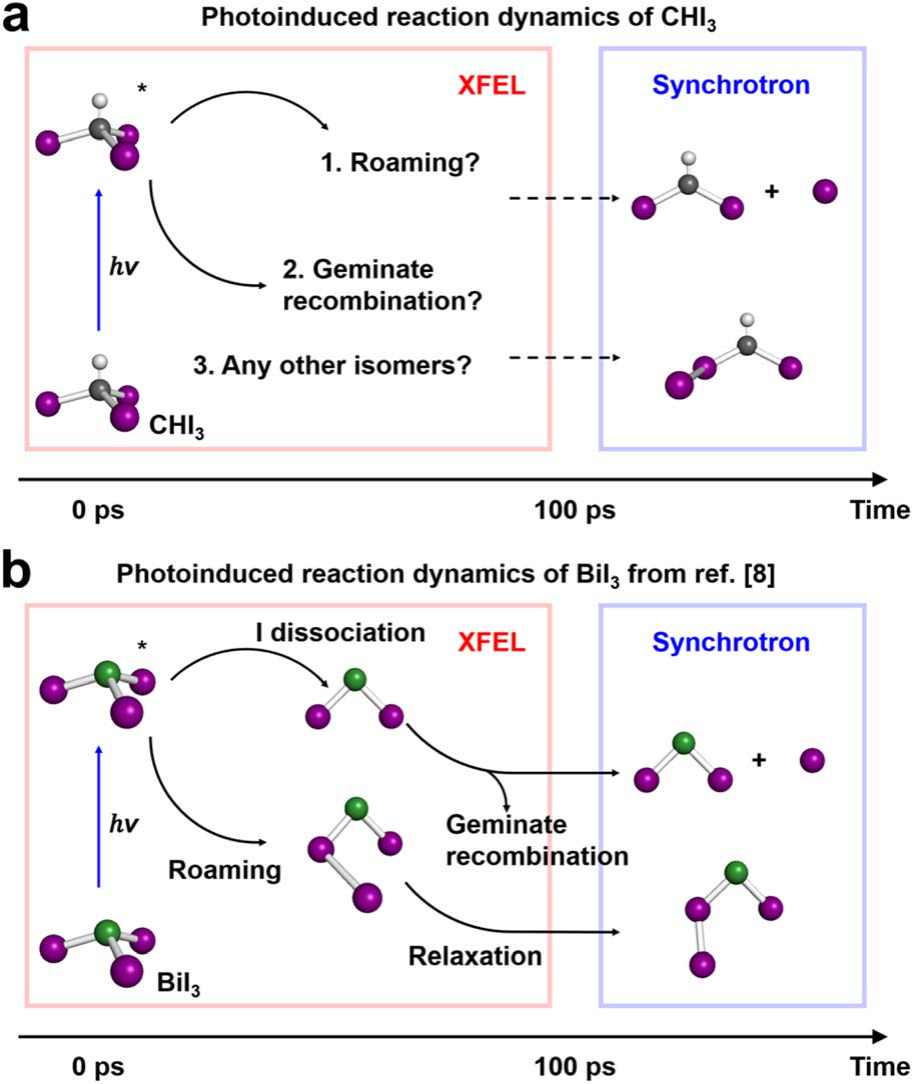
Schematic diagrams for the photoinduced reaction dynamics of CHI_3_ in cyclohexane and BiI_3_ in acetonitrile. (a) The photoinduced reaction dynamics of CHI_3_ in cyclohexane. Two intermediates present after 100 ps had been revealed by a previously conducted TRXL experiment at a synchrotron facility. Three major questions that can be answered by conducting an fs-TRXL experiment at an XFEL facility are: (1) whether roaming reaction occurs, (2) whether geminate recombination occurs, and (3) whether there are any other isomers. (b) The photoinduced reaction dynamics of BiI_3_ in acetonitrile. BiI_3_ shows distinct three pathways: (1) roaming-mediated early isomer formation, (2) geminate recombination of radicals, and (3) late isomer formation *via* relaxation of early isomer. In both (a) and (b), all chemical species are illustrated with colored spheres representing each element: white (H), grey (C), purple (I), and green (Bi).

CHI_3_, along with CH_2_I_2_, is a substance used in the photo-cyclopropanation of olefins and is representative of polyhalomethanes in research.^9–11^ As a triiodo-molecular system, CHI_3_ resembles BiI_3_, showing valence isoelectronicity, and serves as an appropriate comparative molecular system for observing isomerization mechanisms. Previous studies have revealed the existence of *iso*-CHI_2_–I during the photolysis process of CHI_3_ in cyclohexane.^12–15^ However, capturing the isomerization process of CHI_3_*via* time-resolved X-ray liquidography (TRXL)^16–21^ in third-generation synchrotrons posed challenges due to limited temporal resolution, typically limited to approximately 100 picoseconds.^22^ By this time point, both isomer and radical intermediates were already coexisting, and the initial processes leading to the formation of each intermediate could not be identified. Fortunately, the development of X-ray free-electron laser (XFEL) facilities enabled us to utilize femtosecond time resolution X-ray sources with more intense X-ray pulses. This breakthrough allowed us to directly observe the iodine dissociation process and the formation of the isomer in the sub-picosecond temporal regime (Fig. 1).

In this study, we present the results of our investigation into the photoinduced reaction dynamics of CHI_3_ in cyclohexane using femtosecond time-resolved X-ray liquidography (fs-TRXL). Specifically, we focus on the ultrafast structural dynamics during C–I bond cleavage and the formation of *iso*-CHI_2_–I through geminate recombination of radical species. We also analyzed the anisotropic signal component generated by the photoselective excitation caused by linearly polarized pump pulses, resolving its decay kinetics and structural origin. Furthermore, we draw comparisons with BiI_3_, which exhibits roaming-mediated formation of an early isomer. Despite the similarities in structure and number of valence electrons, the photolysis of CHI_3_, unlike that of BiI_3_, did not result in the roaming-mediated formation of an early isomer. This comparison highlights significant differences in the photoinduced reaction dynamics of CHI_3_ and BiI_3_, which are likely attributed to differences in the properties of the central moieties, carbon hydride and bismuth.

## Results and discussion

### Experimental data, ΔS(*q*, *t*) of fs-TRXL

**Fig. 2:**
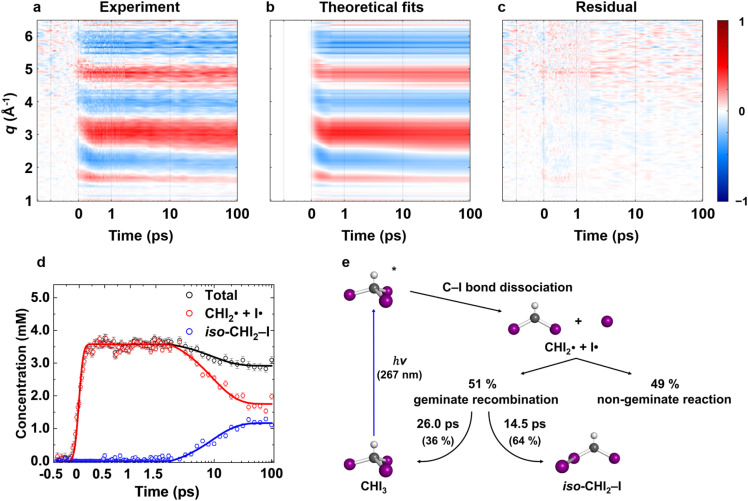
Isotropic fs-TRXL signals of CHI_3_ with theoretical fit results, and the corresponding concentration profiles and kinetic model. (a) Experimental data *q*ΔS^⊥^_0_(*q*, *t*) plotted in a contour map with time (ps) and *q* (Å^−1^) as *x*- and *y*-axis, respectively. (b) Corresponding theoretical fit results derived from the structural analysis and the exponential kinetics model. (c) Residual obtained by subtracting theoretical fit results from the corresponding experimental data. All panels share a common color scale representing the amplitude of the signal in arbitrary unit on the very right side. (d) The concentration profiles of the intermediate species. (e) Kinetic model of CHI_3_ photodissociation process. A more comprehensive version is in Fig. S7.† The structural refinement was applied for the early time period (*t* ≤ 500 fs), and the LCF was used for the later time period (*t* > 500 fs). The results from these two approaches are stitched together and shown in (b). In (d), the plots are presented in a linear time scale up to 1.8 ps, then are presented in a logarithmic time scale extending to 100 ps. The kinetic model fit results (solid lines) well describe the linear combination fit results (dots with one-standard-deviation error bars). The decrease in both total and radical concentrations is clearly shown in (d).

We obtained the difference scattering patterns of photoexcited CHI_3_ in cyclohexane for various time delays. The obtained difference scattering patterns are decomposed into isotropic (ΔS_0_(*q*, *t*)) and anisotropic (ΔS_2_(*q*, *t*)) components *via* an established method.^23,24^ The isotropic signal components are further processed *via* the projection to extract the perpendicular component (PEPC)^25^ method to remove the kinetic contributions of solvent heating and experimental artifacts arising from the fluctuation of experimental conditions, such as the fluctuation in the thickness of sample and X-ray intensity. The resulting PEPC-treated signal is denoted as ΔS^⊥^_0_(*q*, *t*), where the symbol ⊥ indicates PEPC-treated data (see ESI† for details). *q*ΔS^⊥^_0_(*q*, *t*), ΔS^⊥^_0_(*q*, *t*) multiplied by *q* to emphasize the signal in high-*q* region, is depicted in Fig. 2a as a contour plot. For the time delays less than 500 fs, a sudden rise and peak shift are observed, indicating the ultrafast structural dynamics during C–I bond cleavage. These changes are well-resolved with the temporal resolution of the experiment, which is represented by the instrument response function (IRF) and determined to be approximately 180 fs (180 ± 7 fs). After *t* = 500 fs, slow and smooth changes in signals are observed, suggesting that the initially formed intermediates undergo further reactions.

### Overall reaction pathways and kinetics of photoexcited CHI_3_

First, we examined whether the data can be well described by only the two intermediates (
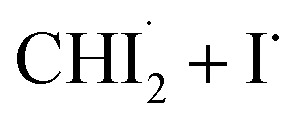
 and *iso*-CHI_2_–I) that were reported to be populated at time delays longer than 100 ps *via* a TRXL study at a synchrotron.^22^ For this purpose, we performed linear combination fitting (LCF) analysis, where the data at each time delay is fitted with a linear combination of basis components. Specifically, species-associated difference scattering curves (SADSs) corresponding to the two intermediates were PEPC-treated in the same manner as the experimental data. The PEPC-treated SADSs were then used to fit the experimental data *via* linear combination. The solute structures used to calculate the SADSs, namely those for the species CHI_3_, *iso*-CHI_2_–I, and CHI_2_, were adapted from experimentally refined structures reported in the previous study.^22^ If the data is well described by the linear combination of the known solute components, it confirms that no other intermediates beyond the known ones contribute to the reaction pathway. Conversely, if the fit is unsatisfactory, it indicates the presence of additional short-lived intermediates significantly contributing to the signal, beyond the known intermediates. The results of the LCF analysis are presented in Fig. S1.† It is observed that the data after 500 fs time delay is well described by a combination of signals corresponding to the two intermediates. This confirms that, at least after the 500 fs time delay, there are no additional short-lived intermediates.

Through the LCF analysis, we extracted how the contributions of 
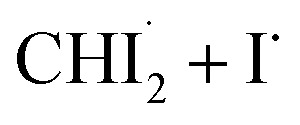
 and *iso*-CHI_2_–I vary over time, revealing the concentration changes of each species. This information is depicted in Fig. 2d (empty circles). The following insights can be gleaned upon examination of the data. At early time points (<1.5 ps), radicals 
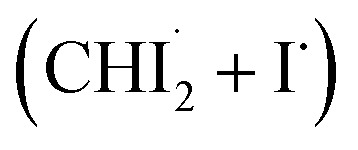
 are formed first. During this period, the contribution of radicals is dominant, while the contribution of the isomer is negligible. Subsequently, between 2 ps and 40 ps, a decrease in the concentration of 
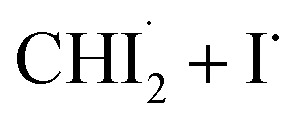
 is observed, accompanied by an increase in the concentration of *iso*-CHI_2_–I. It is apparent that the time delay of 40 ps is too early for 
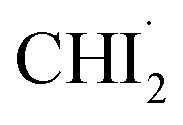
 and I˙ to undergo a non-geminate bimolecular recombination.^26–28^ Therefore, it can be assigned that during this time domain, 
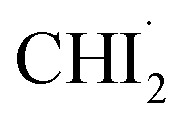
 and I˙ radicals geminately recombined. An important observation here is that the molar amount of 
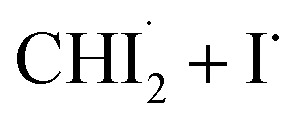
 reduced during this time range is not equivalent to the molar amount of generated *iso*-CHI_2_–I. In Fig. 2d, it is evident that the sum of the concentrations of 
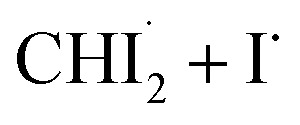
 and *iso*-CHI_2_–I, plotted alongside the concentrations of each species, decreases. This result indicates that not all 
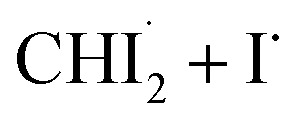
 are converted into *iso*-CHI_2_–I; only a certain fraction undergoes conversion, while the remaining portion either recovers into the parent species, CHI_3_, or remains unchanged. As the time domain of ∼40 ps is too early for a non-geminate recombination, as mentioned earlier, the corresponding recombination of 
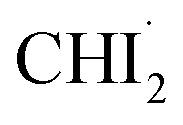
 and I˙ to CHI_3_ should also be a geminate recombination process.

In brief, the concentration changes of each species, as determined through the LCF analysis, indicate the presence of the following two reaction pathways: (1) 
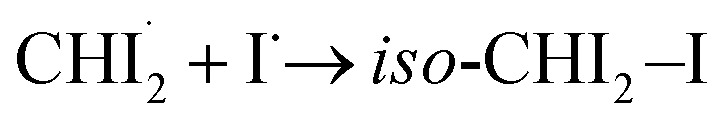
 and (2) 
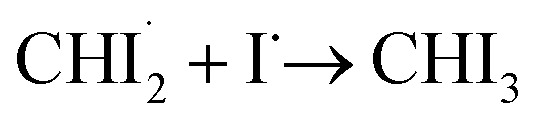
. Before initiating the two pathways, 
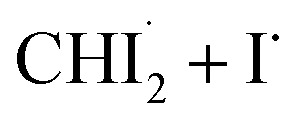
 undergoes a concentration-maintaining period referred to as an induction period. In this phase, the dissociated I radical remains unreactive until it undergoes geminate recombination. Fig. S2† illustrates the comparison between kinetic models with and without the induction period. Incorporating the induction period into the kinetic model yields time constants of (1) 14.5 ps and (2) 26.0 ps, providing a more accurate depiction of the experimental data than that without the induction period. In contrast, when the induction period is ignored, the kinetic model extends the time constants to account for the persistent nature of radical concentrations in the early time domain (<1.8 ps), leading to much larger time constants of 22.6 ps and 39.3 ps for the pathways (1) and (2), respectively. The absence of the induction period in the kinetic model exacerbates discrepancies, particularly in the 1.8 ps to 10 ps region (see Fig. S2c and d†). Based on these, we established the optimal kinetic model for the photoinduced reaction dynamics of CHI_3_, comprising (1) the amount of initially generated radicals from photoexcited CHI_3_, (2) the induction period of radicals, (3) the fraction of radicals involved in secondary geminate recombination, and (4, 5) two time constants *τ*_1_ and *τ*_2_ for each pathway described earlier, in total of five parameters.

In the best-fit results, 5.2 ± 0.6 mM of CHI_3_ out of 20 mM is initially excited by the pump pulse. Of this, 1.6 ± 0.2 mM of CHI_3_ relaxes back to the ground state, while the remaining 3.6 ± 0.4 mM undergoes C–I bond cleavage, generating 
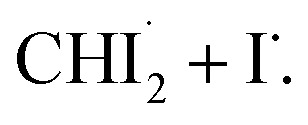
 Among the initially generated 3.6 mM of 
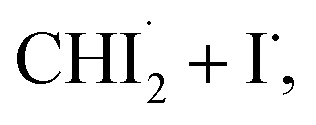
 51 ± 1.1% (1.8 mM) undergo geminate recombination after a 1.8 ps of induction period, while the remaining 49 ± 1.1% (1.8 mM) persist unchanged until 100 ps. The reactive radicals (mentioned earlier, the 51%) are further subdivided, with 64 ± 2.3% (1.2 mM) undergoing isomerization to produce *iso*-CHI_2_–I with a time constant, *τ*_1_ = 14.5 ± 1.0 ps, and the remaining 36 ± 2.3% (0.65 mM) relaxing back to their parent molecule, CHI_3_, with a time constant, *τ*_2_ = 26.0 ± 1.9 ps. The ratio of recombination to the isomer and to the parent molecule, 64 : 36, can be further supported by employing the random collision model between 
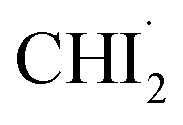
 and I˙. In the model, the collision of 
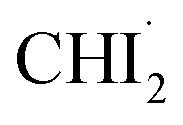
 and 
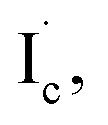
 where the subscript c is used to indicate the dissociated I atom among three I atoms of CHI_3_, can result in the production of *iso*-CHI_2_–I with two targets (I_a_ and I_b_, two iodine atoms in 
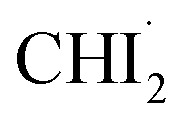
) or CHI_3_ with one target (the carbon atom), in total of three targets (I_a_, I_b_, and C). The ratio between the newly formed *iso*-CHI_2_–I *vs.* CHI_3_ in this simplified model is 2 : 1 = 67 : 33, which is almost identical to the one obtained from the kinetic model (64 : 36). The two time constants, *τ*_1_ = 14.5 ps and *τ*_2_ = 26.0 ps, yield an apparent time constant, *τ* = 9.3 ± 1.1 ps, which aligns closely with the time constant, *τ* = 9.6 ± 0.6 ps, obtained from the results of the SVD analysis (see Fig. S3d†). Here, an “apparent” time constant refers to a simplified kinetic behavior that is observed in the data and represents the combined effect of two independent processes, each with distinct time constants *τ*_1_ and *τ*_2_. When these two processes contribute simultaneously to the observed kinetics, the apparent time constant can be calculated using the relationship: 1/*τ* = 1/*τ*_1_ + 1/*τ*_2_. This equation is derived from the principle that the overall rate of change (1/*τ*) is the sum of the rates (1/*τ*_1_ and 1/*τ*_2_) of the two independent processes. The combined result of these processes can be expressed through the apparent time constant, which provides a simplified but meaningful representation of the system's overall kinetics.

The unchanged, remaining radicals (the 49%) undergo slow, non-geminate recombination after 100 ps, as already revealed in the previous TRXL study. The ratio between the reactive radicals and the remaining radicals in CHI_3_ (51 : 49) is similar to that observed in other triiodides, I_3_^−^ (58 : 42)^29^ and BiI_3_ (60 : 40),^8^ but slightly smaller. The ratio is influenced by the characteristics of the solvent and solute system; future experiments in different solvent systems, such as CHI_3_ in methanol, could provide further confirmation.

The kinetic analysis of the TRXL data shows that the recombination of CHI_2_ and I radicals occurs over a timescale of several to tens of picoseconds, with specific time constants of 14.5 ps and 26.0 ps. Based on these time constants, we conclude that this can be interpreted as geminate recombination. More specifically, when compared to other systems containing two or three iodine atoms, such as HgI_2_ ^30^ and I_3_^−^,^31^ this process likely corresponds to what is described as secondary geminate recombination in studies reporting the kinetics of those systems. In this process, geminate radicals that escaped the first solvation shell but remained in close proximity eventually recombine. While the lifetime of this radical pair may appear unusually long, similar instances of radical pairs remaining in close proximity for tens to hundreds of picoseconds have been reported in other systems, such as CH_2_I_2_.^32^

It should be noted that the accurate reaction pathways could be determined because the relative concentrations of both species were quantitatively determined *via* TRXL. Achieving such quantitative determination in time-resolved spectroscopic measurements is not trivial. In contrast, TRXL enables these quantitative measurements thanks to the quantitative nature of its signal. In other words, the TRXL signal—from any species—can be accurately simulated, capturing not only the shape but also the precise, absolute amplitude. This quantitative nature of the TRXL signal allows for precise determination of the relative branching ratios of any two species of interest (see Fig. S10†). Such quantitative determination *via* time-resolved spectroscopic signals requires prior knowledge of accurate transition cross-sections for the species of interest. Since such information is not readily available, it is generally estimated through quantum or TD-DFT calculations. However, the oscillator strengths derived from these calculations are often not as accurate as the scattering signals, which can be easily calculated from even roughly predicted molecular structures. This significant advantage of scattering over spectroscopy is frequently overlooked.

### Ultrafast structural dynamics of photoexcited CHI_3_ during early time domain

For the data at time delays earlier than 500 fs, however, the results of the LCF analysis do not match well with the experimental data. This discrepancy is evident in the residuals shown in Fig. S1c.† The shape of the residual continuously changes in *q*-space over time. If the residual originated from a novel, short-lived intermediate species, we would expect the residuals to have a consistent shape, with the amplitude of the residual transiently increasing and decreasing. However, the residuals from the LCF analysis do not exhibit such behavior. This suggests that the residual likely does not originate from the formation and decay of an unknown species, besides 
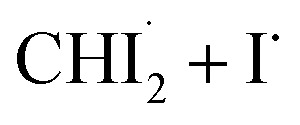
 and *iso*-CHI_2_–I, having a fixed molecular structure, but rather from time-dependent, continuous changes of the molecular structures of the involved species, *i.e.*, a specific wavepacket trajectory. To describe the wavepacket trajectory, we modeled the time-dependent changes of the key structural parameters involved in the process of C–I bond cleavage in CHI_3_, leading to the formation of 
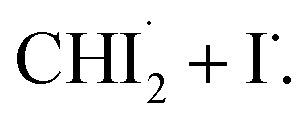
 These key parameters were selected based on the sensitivity plot of CHI_3_. For detailed information, refer to Fig. S4 and ESI.† The resulting wavepacket trajectory, depicted through changes in the representative structural parameters over time delays, is shown in Fig. S5.† The result confirms that the origin of the residual is the ultrafast, continuous structural changes in photoexcited CHI_3_ occurring during C–I bond dissociation process in the early time domain.

### Comparative analysis of triiodine systems with and without roaming-mediated isomer formation

Polyhalomethanes and their relatives, including CHBr_3_ and BiI_3_, exhibit roaming-mediated isomer formation within 200 fs.^7,8^ Notably, BiI_3_, even though it is also one of the triiodides like CHI_3_, demonstrates significant differences compared to CHI_3_ in (1) the presence of early isomer formation through a roaming reaction and (2) the mechanism of (late) isomer generation. Specifically, photolysis of CHI_3_ exclusively yields 
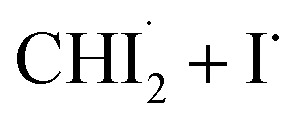
 radicals immediately after the dissociation of I, without involving a roaming reaction. In contrast, in the case of BiI_3_, both roaming-mediated early isomer formation and radical generation through the dissociation of I occur in a 50 : 50 ratio. The rapid formation of the early isomer through roaming reaction, within <200 fs, is known to be solvent-independent^7^ and relies on the interaction between the roaming iodine and the remaining fragment, representing a characteristic of the solute. Therefore, the presence or absence of roaming reaction is predicted to be based on the difference in the central moiety between CHI_3_ and BiI_3_.

For isomer formation, CHI_3_ exclusively undergoes secondary geminate recombination of radicals to form isomer, while BiI_3_ generates the late isomer through the relaxation of the early isomer only. Considering that both cases occur in the fast time regime of a few picoseconds, it is conceivable that the late isomer formation through secondary geminate recombination of radicals could also occur in the BiI_3_ system. However, the absence of such pathway in the previous study is attributed to differences in the central moiety (CH *vs.* Bi) and the solvent (cyclohexane *vs.* acetonitrile) between the two systems, leading to variations in the energy levels of radicals and (late) isomers.

Through further comparison of polyhalomethanes, we observed that halogen identity (for example, Br and I) plays a critical role in influencing roaming dynamics. CH_2_I_2_,^32^ like CHI_3_, contains iodine atoms and does not exhibit roaming-based isomer formation within ∼1 ps, similar to CHI_3_. The structural and behavioral similarities between these iodine-containing systems suggest that the heavier iodine atoms may hinder early-time isomer formation, in contrast to bromine-containing systems like CHBr_3_.^7^ This highlights the significant role that halogen atoms play in determining the dynamics of roaming and recombination in systems with similar central moieties.

### Kinetics of anisotropic signals

**Fig. 3:**
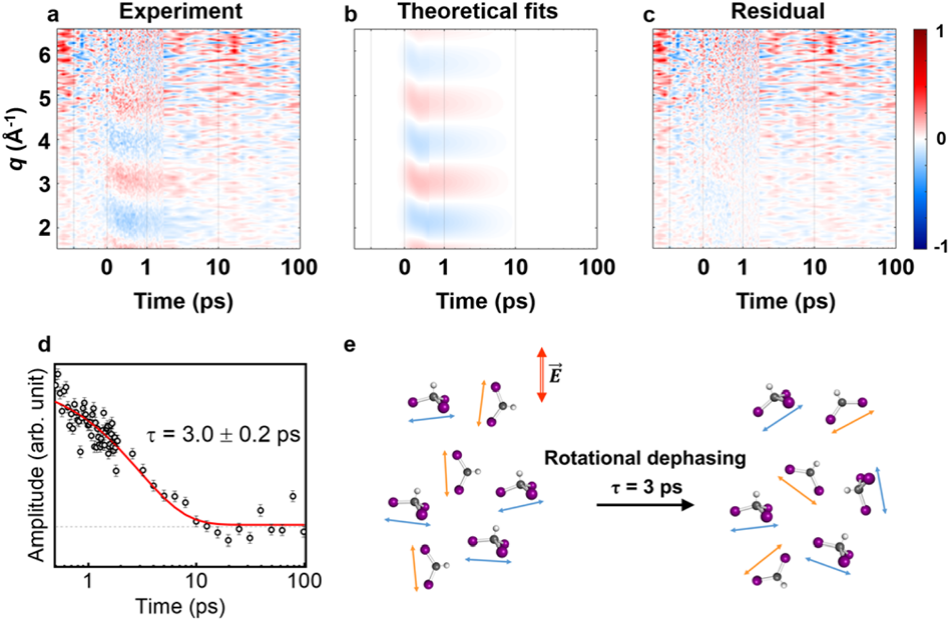
Anisotropic signals aligned with theoretical fit results. (a) Experimental data *q*ΔS_2_(*q*, *t*) plotted in a contour map with time (ps) and *q* (Å^−1^) as *x*- and *y*-axis, respectively. (b) Corresponding theoretical fit results derived from the structural analysis and the exponential kinetics model. (c) Residual obtained by subtracting theoretical fit results from the corresponding experimental data. All panels share a common color scale representing the amplitude of the signal in arbitrary unit on the very right side. (d) The first RSV was fitted by a single exponential function, giving *τ* = 3.0 ± 0.2 ps. (e) The rotational dephasing dynamics of CHI_3_ and 
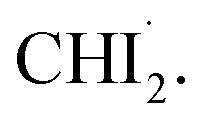

**Fig. 4:**
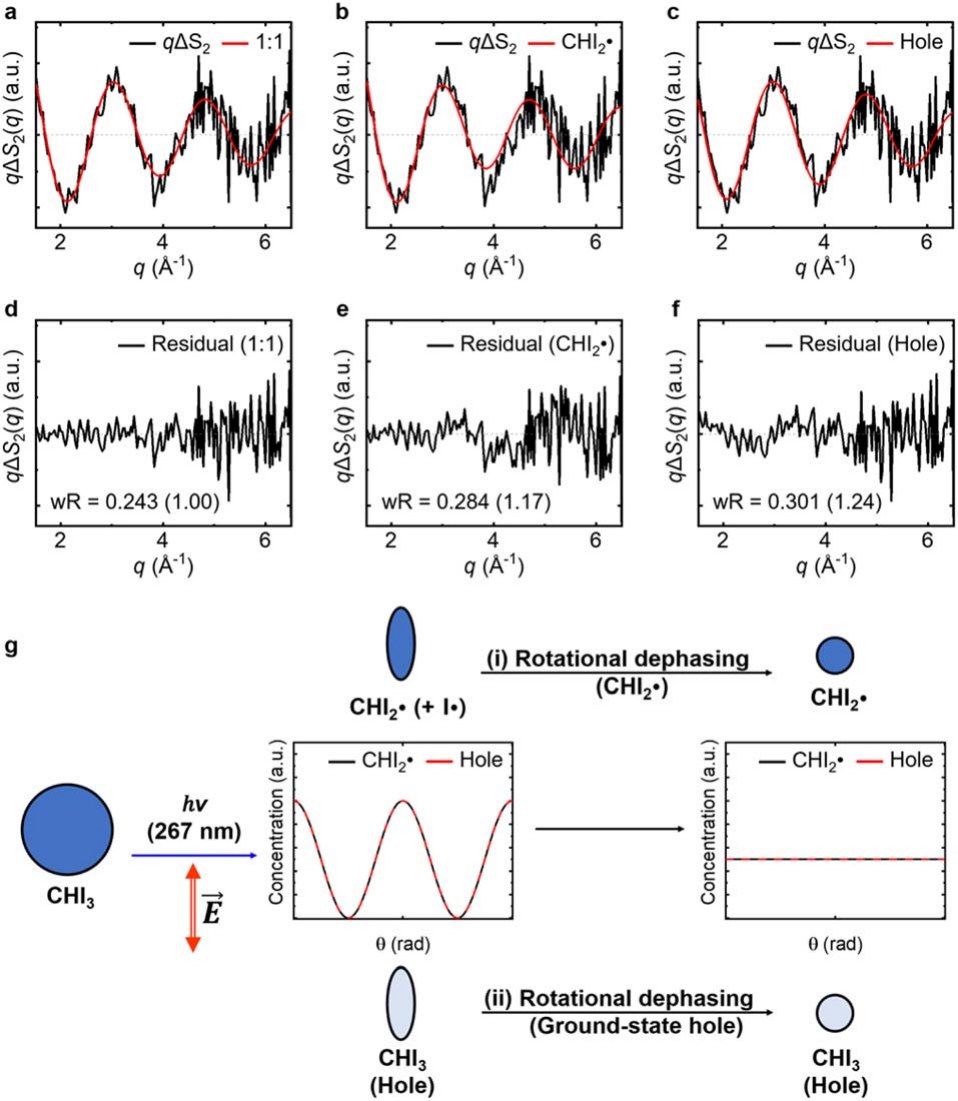
Contribution analysis of the anisotropic experimental data. (a–c) *q*ΔS_2_(*q*) (black), corresponding to the time constant of 3.0 ps, compared to the theoretical anisotropic signals (red) derived from (a) 1 : 1 combination of radicals and ground-state holes, (b) radicals only, and (c) ground-state holes only. (d–f) Residuals of (a)–(c), respectively, obtained by subtracting theoretical anisotropic signals from corresponding *q*ΔS_2_(*q*). The weighted *R*-factors (w*R*) are shown, with the relative ratios compared to the 1 : 1 combination model provided in the parentheses. The residual in (a) is the smallest, suggesting that both radicals and ground-state holes contribute to the anisotropic signals. (g) Schematic diagram illustrating the origin of the anisotropic signals and their kinetics. The blue and pale blue ellipses represent the distributions of the transition dipole moments of 
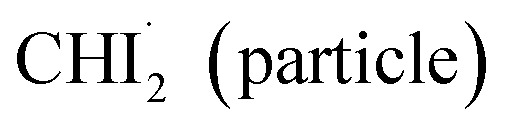
 and CHI_3_ (hole), respectively, which are perturbed by linearly polarized laser pulses depending on the angle (*θ*) between the laser polarization (*E*) and the transition dipole moment. The concentration distributions of 
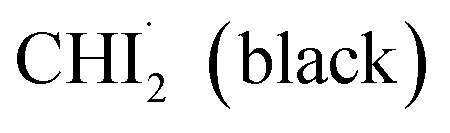
 and CHI_3_ hole with respect to *θ* are plotted in black and red lines, respectively. The 1 : 1 combination model (a) encompasses both pathways, (i) rotational dephasing of the generated 
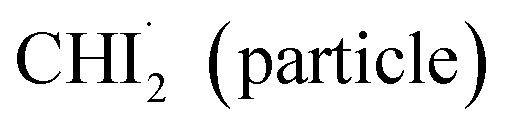
 and (ii) rotational dephasing of depleted CHI_3_ (hole). The radicals-only model (b) involves only the pathway (i), and the ground-state hole-only model (c) involves only the pathway (ii).

As previously mentioned, we aimed to analyze the anisotropic signal isolated from the experimental data in a manner similar to the isotropic signal analysis to confirm the kinetics of the anisotropy. Fig. 3a presents a contour plot of *q*ΔS_2_(*q*, *t*). For time delays less than 500 fs, the ultrafast structural dynamics during C–I bond cleavage induced a peak-shifting feature, as observed in the isotropic signal. This feature was well described by the theoretical anisotropic signal calculated for 
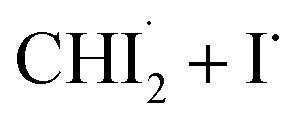
 formation *via* C–I dissociation process, which aligns with the structural dynamics derived from the isotropic signal analysis. For time delays beyond 500 fs, a single major decaying component with a time constant of *τ* = 3.0 ± 0.2 ps was identified through an SVD analysis (see Fig. S6† and 3d). As described in the previous section, the time constants for the radicals returning to either the isomer or CHI_3_ are 14.5 ps and 26.0 ps, respectively, indicating a negligible amount of isomer formation around the 3 ps time range. Therefore, during the time period relevant to the observed anisotropic time constant, the primary contributors to the anisotropic signal are 
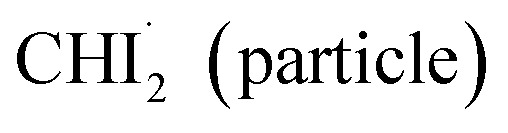
 and depleted CHI_3_ (hole). Based on these considerations, the origin of the anisotropic signal can be explained as follows. Before irradiation, the transition dipole moments of CHI_3_ in its ground state are evenly distributed. Linearly polarized light selectively excites CHI_3_ molecules whose transition dipole moments align well with the polarization direction of the light, with the excitation probability proportional to cos^2^ *θ* where *θ* is the angle between the laser polarization (*E*) and the transition dipole moment. As a result, the excited CHI_3_ and the radicals that subsequently form exhibit a specific directionality according to the transition dipole moment. After orientation-selective photoexcitation, the remaining unexcited CHI_3_ molecules exhibit an uneven distribution of transition dipole moments, as the molecules whose transition dipole moments matched the polarization direction of the light have been selectively excited, leaving an uneven distribution among the unexcited population. This leads to an uneven distribution of transition dipole moments in the ground-state holes and particles, which is depicted in Fig. 4g. Through subsequent rotational dephasing, the ground-state holes and particles gradually return to an even distribution, which is observed as a decaying anisotropic signal over several picoseconds. In this study, we based our fitting on the assumption that the anisotropic signal arises from ground-state holes and excited CHI_3_ particles, which subsequently dissociate into CHI_2_ and I radicals. We tested three possible models for the origin of the anisotropic signal: (1) 1 : 1 combination of radicals and ground-state holes, (2) radicals only, and (3) ground-state holes only. Among the models, the 1 : 1 combination model provided the best fit to the experimental data (Fig. 4). Fig. 3e illustrates the rotational dephasing dynamics of the CHI_3_ system during the photodissociation process. The decay time constants of rotational dephasing for the particle and hole, calculated under the assumption of ellipsoidal molecules,^33^ are 2.3 ps and 4.0 ps, respectively. These values are consistent with the experimental decay time constant of *τ* = 3.0 ± 0.2 ps, further supporting the result.

## Methods

### Experimental methods and sample preparation

The femtosecond time-resolved X-ray liquidography (fs-TRXL) experiment was conducted at the XSS beamline of PAL-XFEL (Pohang Accelerator Laboratory X-ray Free-Electron Laser).^34,35^ X-ray pulses, with an energy of 12.7 keV and a temporal width of <50 fs, were delivered at a repetition rate of 30 Hz and focused to a spot approximately 30 μm in diameter. Optical pump pulses had a wavelength of 267 nm, focused to a spot approximately 200 μm in diameter (full width at half maximum, FWHM), resulting in a laser fluence of ∼2.0 mJ mm^−2^ and a temporal width of ∼100 fs at the sample position. A 20 mM CHI_3_ solution in cyclohexane served as a sample solution, alongside an 8.5 mM 4-bromo-4′-(*N*,*N*-diethylamino)-azobenzene solution in cyclohexane for measuring the solvent heating signals. The sample solution was pumped through a quartz capillary nozzle, generating a 100 μm cylindrical jet vertically. Scattering intensities from the two-dimensional patterns were captured by a charge-coupled-device detector (Rayonix MX225-HS, 5760 × 5760 pixels, 39 × 39 μm^2^ per pixel, 4 × 4 binning mode) with a sample-to-detector distance of ∼34 mm. To capture the CHI_3_ photolysis, the scattering images were collected across a wide-range of pump-probe time delays, spanning from −450 fs to 100 ps, with a total of 108 time delays. More details are described in ESI.†

### Data processing and data reduction

The time-resolved difference scattering images were obtained by subtracting the laser-off scattering images from their corresponding laser-on images of the sample solution. To resolve the X-ray intensity jitter issues that often arise at XFEL facilities, we first paired the laser-off and laser-on scattering images with similar intensities. These paired images were then subtracted to obtain difference scattering images, effectively compensating for the X-ray intensity jitter. Subsequently, these difference images were decomposed into isotropic (ΔS_0_(*q*, *t*)) and anisotropic (ΔS_2_(*q*, *t*)) components and further transformed into one-dimensional difference scattering curves. The solvent heating signals were then subtracted from the sample solution data *via* the projection to extract the perpendicular component (PEPC) method, thereby removing solvent heating contributions and experimental artifacts from the dataset. The PEPC-treated data underwent further analysis through the linear combination fitting (LCF) and the singular value decomposition (SVD) to exclusively assess the kinetic contributions of solute. More details are described in ESI.†

### Anisotropic scattering curve calculation

The anisotropic scattering curves of the ground-state holes and particles were calculated using the method described by Biasin *et al.*^24^ and later applied to a triiodide system by Heo *et al.*^29^ In our analysis, we utilized this method, despite it being specifically designed for symmetric top molecules. The relevant equations used in these studies are not inherently applicable to molecules that do not exhibit symmetric top geometry, such as the photoproduct CHI_2_. The deviation from symmetric top geometry in CHI_2_ may introduce minor inaccuracies in retrieving the anisotropic component of the experimental scattering signal and in calculating the theoretical scattering curves corresponding to the radical species. However, we believe these effects are unlikely to substantially impact our results because the extent of inherent discrepancy between the experimental and simulated anisotropic signals obtained through this approach is expected to be negligible, without interfering with the analysis of dephasing kinetics. Additionally, we did not refine the molecular structure using the anisotropic signal; instead, we used the structural information obtained from the isotropic signal analysis directly in the anisotropic signal analysis. This approach was employed to assign the identity of the species contributing to the anisotropic signal. The small expected distortions in the anisotropic signal are not large enough to affect the identification of these contributing species. Heo *et al.*^29^ successfully used this method for a similar non-symmetric triiodide system. Nevertheless, further simulations or derivations may be necessary to fully validate its application to CHI_2_.

### Details of density functional theory calculation

The initial structures and charges of the reactant and candidate intermediates were obtained from density functional theory (DFT) calculations. We used the ωB97X^36^ functional as the DFT exchange–correlation functional, and the calculations were done using the Gaussian 16 package,^37^ including the NBO program^38^ for the charge calculations. For carbon and hydrogen atoms, aug-cc-pVTZ (AVTZ) all-electron basis sets were used. For iodine atoms, dhf-TZVPP small-core relativistic effective core potential (RECP) was used^39^ to consider the scalar relativistic effects. Solvent effects of cyclohexane were implicitly included by applying an integral equation formalism polarizable continuum model (IEFPCM). DFT calculations were performed to obtain the atomic charge distributions required for the MD simulations, with the MD simulation results subsequently used to calculate the cage terms. We followed the procedures outlined in a previous study^22^ for both DFT calculations and MD simulations.

### Weighted *R*-factor

In Fig. 4, the agreement between the experimental anisotropic signal (*q*ΔS_2_(*q*)) and those derived from the three theoretical models (*q*ΔS_2,theo_(*q*)s) was evaluated by the weighted *R*-factor (w*R*):1
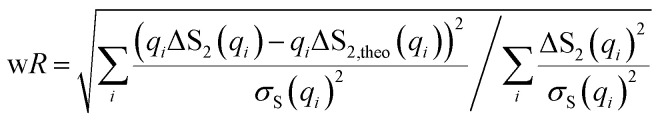
where *σ*_S_ is the standard deviation of the experimental data.

## Conclusions

In summary, we observed the ultrafast structural dynamics of the photodissociation of CHI_3_ in cyclohexane *via* fs-TRXL. Following the iodine dissociation accompanied by structural changes, both *iso*-CHI_2_–I and CHI_3_ are competitively formed through secondary geminate recombination of 
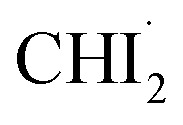
 and I˙ radicals after the induction period. The CHI_3_ system, in contrast to the BiI_3_ system where a roaming-mediated early isomer is generated, only exhibits the formation of radicals, followed by subsequent isomer formation through geminate recombination of them. This disparity between the two systems is attributed to the differences between the two systems, the central moieties (CH *vs.* Bi) and the solvents (cyclohexane *vs.* acetonitrile). Through this paper, we aim to provide insights into whether isomer formation in polyhalomethanes and similar systems is based on geminate recombination or roaming reaction, and further provide clues for controlling the process.

## Author contributions

Conceptualization: H. K., H. I.; data curation: Y. C.; formal analysis: Y. C., H. K.; funding acquisition: H. I.; investigation: Y. L., S. L., J. K., J. H. L., J. K.; methodology: Y. C., H. K., H. I.; software: Y. C., H. K., D. I.; supervision: H. I.; visualization: Y. C.; writing – original draft: Y. C., H. K., H. I.

## Conflicts of interest

There are no conflicts to declare.

## Supplementary Material

SC-015-D4SC04604H-s001

SC-015-D4SC04604H-s002

## Data Availability

The data supporting this article have been included as part of the ESI.†
