# Supplementary material for: The carbon–iodine bond cleavage and isomerization of iodoform visualized with femtosecond X-ray liquidography
Source: Chem Sci. 2024 Oct 29;15(45):19104–12. doi: 10.1039/d4sc04604h (PMC11523838; doi:10.1039/d4sc04604h)
Supplement: SC-015-D4SC04604H-s001 [file SC-015-D4SC04604H-s001.pdf]

Supplementary Information (SI) for Chemical Science.
This journal is © The Royal Society of Chemistry 2024

Supplementary Information

The carbon–iodine bond cleavage and isomerization of iodoform visualized with femtosecond X-ray liquidography

Yongjun Cha,[ab] Hosung Ki,[ab] Donghwan Im,[ab] Yunbeom Lee,[ab] Seonggon Lee,[ab] Jungmin Kim,[ab] Jae Hyuk Lee,[c] Jeongho Kim[d] and Hyotcherl Ihee*[ab]

[a]Department of Chemistry, Korea Advanced Institute of Science and Technology (KAIST), Daejeon 34141, Republic of Korea

[b]Center for Advanced Reaction Dynamics (CARD), Institute for Basic Science (IBS), Daejeon 34141, Republic of Korea

[c]Pohang Accelerator Laboratory, Pohang 37673, Republic of Korea

[d]Department of Chemistry, Inha University, 100 Inha-ro, Michuhol-gu, Incheon 22212, Republic of Korea

*Corresponding author. E-mail: hyotcherl.ihee@kaist.ac.kr

Table of Contents

Supplementary Methods **Pages**

1. Femtosecond time-resolved X-ray liquidography (fs-TRXL) experiment············ S3
2. Generation of time-resolved difference scattering curves ································· S4
3. Linear combination fitting of $\Delta S(q, t)$ ··· S5
4. Singular value decomposition of $\Delta S(q, t)$········ ··· S5
5. Structural analysis on ΔS_0 and ΔS_2 (q, $t > 500$ fs)·· S6
6. Structural analysis on ΔS_0 and ΔS_2 (q, $t \leq 500$ fs) ·· S6 – S7
7. Molecular dynamics simulation ··· S7

Supplementary Figures **Pages**

1. Figure S1 ·· S8
2. Figure S2 ·· S9
3. Figure S3 ·· S10
4. Figure S4 ·· S11
5. Figure S5 ·· S12
6. Figure S6 ·· S13
7. Figure S7 ·· S14
8. Figure S8 ·· S15
9. Figure S9 ·· S16
10. Figure S10 ·· S17
11. Figure S11 ·· S18
12. Figure S12 ·· S19

References **Pages**

1. References ·· S20

Supplementary Methods

1. Femtosecond time-resolved X-ray liquidography (fs-TRXL) experiment

The femtosecond time-resolved X-ray liquidography (fs-TRXL) experiment was conducted at the XSS beamline of PAL-XFEL (Pohang Accelerator Laboratory X-ray Free-Electron Laser).[1,2] X-ray pulses, with an energy of 12.7 keV and a temporal width of ~30 fs, were delivered at a repetition rate of 30 Hz and focused to a spot approximately 30 μm in diameter. Optical pump pulses, generated via third-harmonic generation of 800 nm femtosecond laser pulses from a Ti:sapphire regenerative amplifier, had a wavelength of 267 nm. These pulses were then spatially focused to a spot approximately 200 μm in diameter (full width at half maximum, FWHM), resulting in a laser fluence of ~2.0 mJ/mm^2 and a temporal width of ~100 fs at the sample position. The laser and X-ray beams were overlapped at the sample position with a crossing angle of 10°, yielding an experimental instrument response function (IRF) of 180 fs. This IRF value derived from the analysis of experimental data closely matches the estimated value based on the experimental conditions. This estimation considers the convolution of several factors: the temporal pulse widths of the X-ray and optical laser pulses (30 fs and 100 fs, respectively), their velocity mismatch when passing through the 100 μm liquid jet (166 fs), and the timing jitter between the X-ray and optical laser pulses (25 fs). The convolution yields a value of 198 fs, which closely matches the experimentally determined value of 180 fs. A 20 mM CHI_3 (Aldrich, 99.9%) solution in cyclohexane served as a sample solution, alongside an 8.5 mM 4-bromo-4′-(N,N-diethylamino)-azobenzene (HANCHEM, 99.9%) solution in cyclohexane for measuring the solvent heating signals. All reagents were used without further purification. The sample solution was pumped through a quartz capillary nozzle, generating a 100 μm cylindrical jet vertically. Scattering intensities from the two-dimensional patterns were captured by a charge-coupled-device detector (Rayonix MX225-HS, 5760 × 5760 pixels, 39 × 39 μm^2 per pixel, 4 × 4 binning mode) with a sample-to-detector distance of ~34 mm. To capture the CHI_3 photolysis, the scattering images were collected across a wide range of pump-probe time delays, spanning from -450 fs to 100 ps, with a total of 108 time delays. These included intervals from -450 fs to 1.8 ps (at increments of 25 fs), as well as specific time points at 2.51 ps, 3.16 ps, 3.98 ps, 5.01 ps, 6.31 ps, 7.94 ps, 10 ps, 12.6 ps, 15.8 ps, 20.0 ps, 25.1 ps, 31.6 ps, 39.8 ps, 50.1 ps, 63.1 ps, 79.4 ps, and 100 ps. Laser-off images at a time delay of -3 ps were acquired before capturing each laser-on image.

2. Generation of time-resolved difference scattering curves

The time-resolved difference scattering images were obtained by subtracting the laser-off scattering images from their corresponding laser-on images of the sample solution. Subsequently, these difference images were decomposed into isotropic ($\Delta S_0(q, t)$) and anisotropic ($\Delta S_2(q, t)$) components and further transformed into one-dimensional difference scattering curves via an established method.[3,4] Simply, two-dimensional difference images can be decomposed as follows:

$$\Delta S(q,t,\theta) = \Delta S_0(q,t) - ((3\cos^2\theta - 1)/2)\Delta S_2(q,t) \quad (1)$$

where q is the momentum transfer vector, t is the time delay, and θ is the angle between the laser polarization axis and q. Difference images and 1D difference scattering curves of the dye solution were generated in the same manner to obtain the solvent heating signals. These signals were then extracted from the curves and subtracted from the sample solution data via the projection to extract the perpendicular component (PEPC)[5] method, thereby removing solvent heating contributions and experimental artifacts from the dataset. The PEPC-treated data underwent further analysis through the linear combination fitting (LCF) and the singular value decomposition (SVD) to exclusively assess the kinetic contributions of solute. The raw experimental data and its individual components—artifacts, heating signals, and solute-related signals—are presented in Fig. S8 and Fig. S9, respectively. Fig. S12 presents the raw experimental data alongside its heat and artifact-filtered versions, including the PEPC-treated data, all displayed as contour maps.

The isotropic component, $\Delta S_0(q, t)$ was PEPC-treated with isotropic solvent heating signals, resulting in $\Delta S_0^{\perp}(q,t)$. The anisotropic component $\Delta S_2(q, t)$, on the other hand, required a different approach. The anisotropic solvent heating signals, primarily originating from the optical Kerr effect near the time-zero region, exhibited a small signal at the low-q region and noisy features at the high-q region. Simply applying the PEPC method to the data with noisy signals could degrade its quality. Thus, to remove the contribution of the anisotropic solvent heating signals, a small portion of the low-q region (q = 1.0 – 1.5 Å^{-1}, 18 points out of 185 points) was removed, resulting in $\Delta S_2'(q, t)$, denoted by the symbol ′ indicating the removal of the low-q region.

3. Linear combination fitting of $\Delta S(q, t)$

In a linear combination fitting (LCF) analysis, experimental data ($\Delta S_{exp}(q, t)$) is fitted against a sum of theoretical curves multiplied by their contributions ($\Sigma\alpha\Delta S_{theo}(q, t)$) by minimizing χ^2 value:

$$\chi^2 = (\Delta S_{exp}(q,t) - \sum \alpha_i \Delta S_{theo,i}(q,t)) / \sigma^2 \quad (2)$$

where σ is the standard deviation of the experimental data. Here, the contributions of the theoretical curves are proportional to the concentration of the corresponding species, thereby imposing the constraint, $\alpha_i \geq 0$. We applied the linear combination fitting (LCF) analysis to the PEPC-treated isotropic ($\Delta S_0^{\perp}(q,t)$) signals based on the knowledge of 100 ps structural dynamics from the previous TRXL study,[6] which unveiled two major intermediates at 100 ps, radicals (CHI_2• and I•) and isomer (*iso*-CHI_2–I). Theoretical isotropic signals of the two intermediates were generated by adding solute terms obtained from the Debye equation and cage terms obtained from MD simulations.[7] The signals were further PEPC-treated to be fairly compared to $\Delta S_0^{\perp}(q,t)$, since PEPC-treating might deform the shape of the curves while preserving their temporal contributions. The results of the LCF analysis are presented in Fig. S1 and further used to determine dynamics for $t > 500$ fs (see Supplementary Method 5 for details).

4. Singular value decomposition of $\Delta S(q, t)$

We employed the singular value decomposition (SVD) analysis on the PEPC-treated isotropic ($\Delta S_0^{\perp}(q,t)$) and modified anisotropic ($\Delta S_2'(q, t)$) signals to extract the major components of the scattering curve and their corresponding temporal profiles. A detailed explanation of the SVD analysis and related expressions can be found in a previous study.[8] In this work, $\Delta S_0^{\perp}(q,t)$ is an $n_q \times n_t = 185 \times 108$ matrix and $\Delta S_2'(q, t)$ is a 167 × 108 matrix. The results of the SVD analysis are presented in Fig. S3 ($\Delta S_0^{\perp}(q,t)$) and Fig. S6 ($\Delta S_2'(q, t)$). In Fig. S3, three major components are identified: two (RSV_1, RSV_2) across all time delays and one (RSV_3) near time-zero region. The former two, encompassing information on the exponential kinetics of intermediate populations, were fitted by single exponential functions with a time constant of $\tau = 9.6 \pm 0.6$ ps. In Fig. S6, one major component demonstrates dynamics with a time constant of $\tau = 3.0 \pm 0.2$ ps, well-explained by rotational dephasing of particle and ground-state hole (see Figs. 3, 4 and Supplementary Method 6 for details).

5. Structural analysis on ΔS_0 and ΔS_2 (q, $t > 500$ fs)

As briefly mentioned in Supplementary Method 3, we conducted the LCF analysis of $\Delta S_0^{\perp}(q,t)$ using PEPC-treated theoretical signals of the known intermediates (CHI_2• + I• and *iso*-CHI_2–I). This enabled us to 1) validate previously reported intermediates and 2) identify the point at which the data can be explained by changes in the concentrations of known species. Fig. S1 presents the results of the LCF analysis as a contour map, demonstrating a region ($t >$ 500 fs) where residuals are negligible. This suggests that the known intermediates, radicals (CHI_2• and I•) and isomer (*iso*-CHI_2–I), along with their time-dependent concentration profiles adequately account for this portion of the data without requiring further structural changes. In Fig. S2, the LCF analysis result is well-described by the kinetic model with time constants of τ_1 = 14.5 ps and τ_2 = 26.0 ps. The kinetic model gives an apparent time constant of τ = 9.3 ps, which is comparable to the time constant of τ = 9.6 ps obtained from the SVD analysis, showing consistency between the two analyses.

Fig. 3d and Fig. S6 present the SVD analysis results of $\Delta S_2'(q, t)$, revealing a major anisotropic component with a decaying time constant of $\tau = 3.0 \pm 0.2$ ps. Anisotropic signals are generated by the linearly polarized pump pulse and decay through rotational dephasing of CHI_2• (particle) and depleted CHI_3 (hole), as described in Fig. 4g. Three plausible models were compared in Fig. 4, and model (1), the 1:1 combination model of particle and hole, with the least residual, was selected as the best model.

6. Structural analysis on ΔS_0 and ΔS_2 (q, $t \leq 500$ fs)

As briefly mentioned in the main text, in the region where $t \leq 500$ fs, a sudden rise and peak shift is observed that cannot be fully explained by theoretical signals from the known intermediates (see Fig. S1). As described in Supplementary Method 5, only radicals are present at t = 500 fs, indicating that the formation of CHI_2• + I• radicals from CHI_3 via C–I dissociation occurs between t = 0 fs and t = 500 fs, along with the corresponding wavepacket trajectory.

Based on the sensitivity plot of the CHI_3 system which highlights major structural parameters of the molecule (see Fig. S4 and Fig. S11), we selected (1) the I_a–I_b distance, (2) the $I_{a,b}$–I_c distance, and (3) the Debye-Waller factor (DWF) of (2) as fitting parameters. Here, I_a and I_b represent the iodines of the CHI_2• fraction, I_c is an iodine that becomes I•, and $I_{a,b}$ is the center point between I_a and I_b. The results of the fitting are illustrated in Fig. S5, in plots depicting time vs. parameters.

For the anisotropic signal, the same wavepacket trajectory fitting was performed

alongside the isotropic signal, minimizing residual. The fitting result is illustrated in Fig. 3.

7. Molecular dynamics simulation

Molecular dynamics (MD) simulations were performed using the MOLDY[9] program to obtain the cage terms for all the chemical species involved in the reaction. One rigid solute molecule was embedded in a virtual cubic box of ~26 Å size containing 256 rigid cyclohexane solvent molecules. The internal structure of each molecule was fixed, and the intermolecular interactions were governed by Coulomb forces and Lennard-Jones potentials. For describing the intermolecular interactions, we used a twelve-site all-atom (AA) model of cyclohexane, OPLS-AA force field.[10] Structures and charges of solute molecules (CHI_3, *iso*-CHI_2–I, CHI_2•, and I•) were optimized using the DFT method. All simulations were performed at an ambient temperature of 300 K with a solvent density of 0.779 g/cm^3. To obtain the theoretical heating signals of cyclohexane, two virtual cubic boxes of ~45 Å size containing 512 rigid cyclohexane solvent molecules at 300 K and 330 K were simulated.

Supplementary Figures

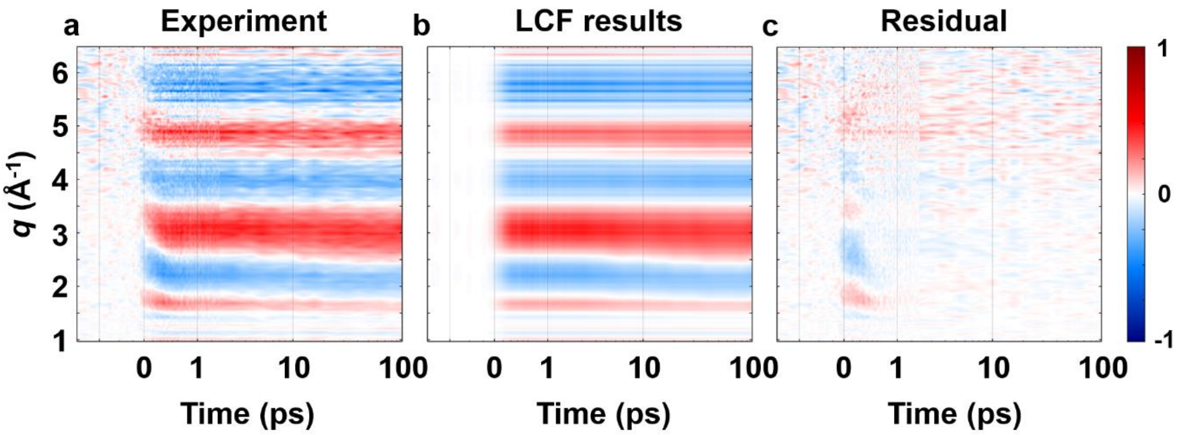

Figure S1. Isotropic experimental data and its LCF fit. (a) Experimental data $q\Delta S_0^{\perp}(q,t)$ plotted in a contour map with time (ps) and q (Å^{-1}) as x- and y-axis, respectively. (b) Corresponding linear combination fitting (LCF) results using species-associated difference scattering signals corresponding to the two intermediates as basis components. (c) Residual obtained by subtracting LCF results from the corresponding experimental data. In (c), residuals of experimental signals near time zero which cannot be solely explained by two known intermediates are clearly shown. All panels share a common color scale representing the amplitude of the signal in arbitrary unit on the very right side.

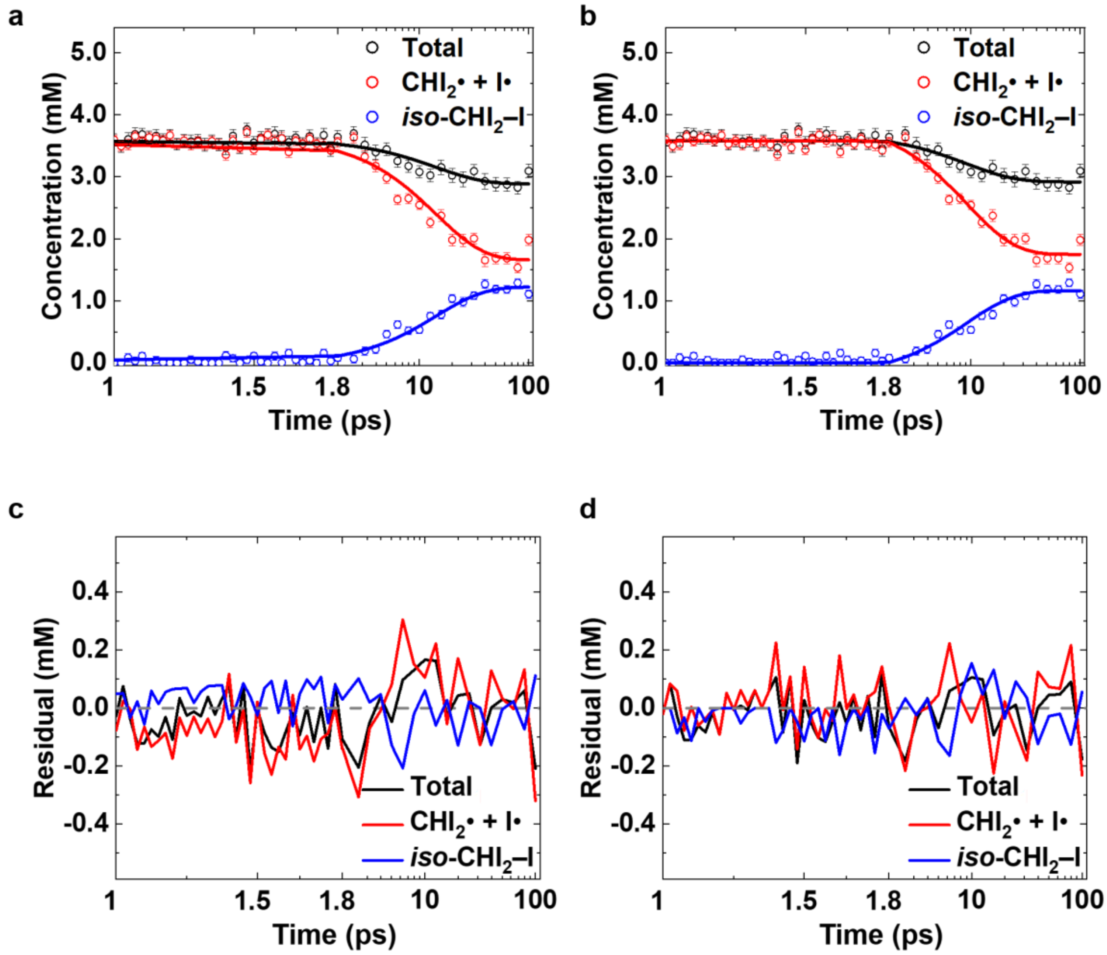

Figure S2. Kinetic analysis of the experimental data after 1 ps. (a, b) Kinetic models with (a) no induction period and (b) 1.8 ps compared to the LCF fit results. (c, d) Residuals of (a) and (b), respectively, obtained by subtracting LCF fit results from corresponding kinetic model fit results. The kinetic models are composed of two single exponential decay functions with time constants τ_1 and τ_2, respectively. In (a), the kinetic model with $\tau_1 = 22.6$ ps and $\tau_2 = 39.3$ ps gives an apparent time constant $\tau = 14.4$ ps. In (b), $\tau_1 = 14.5$ ps and $\tau_2 = 26.0$ ps, giving $\tau = 9.3$ ps, which well matched the results from the LCF and the SVD analysis ($\tau = 9.6$ ps).

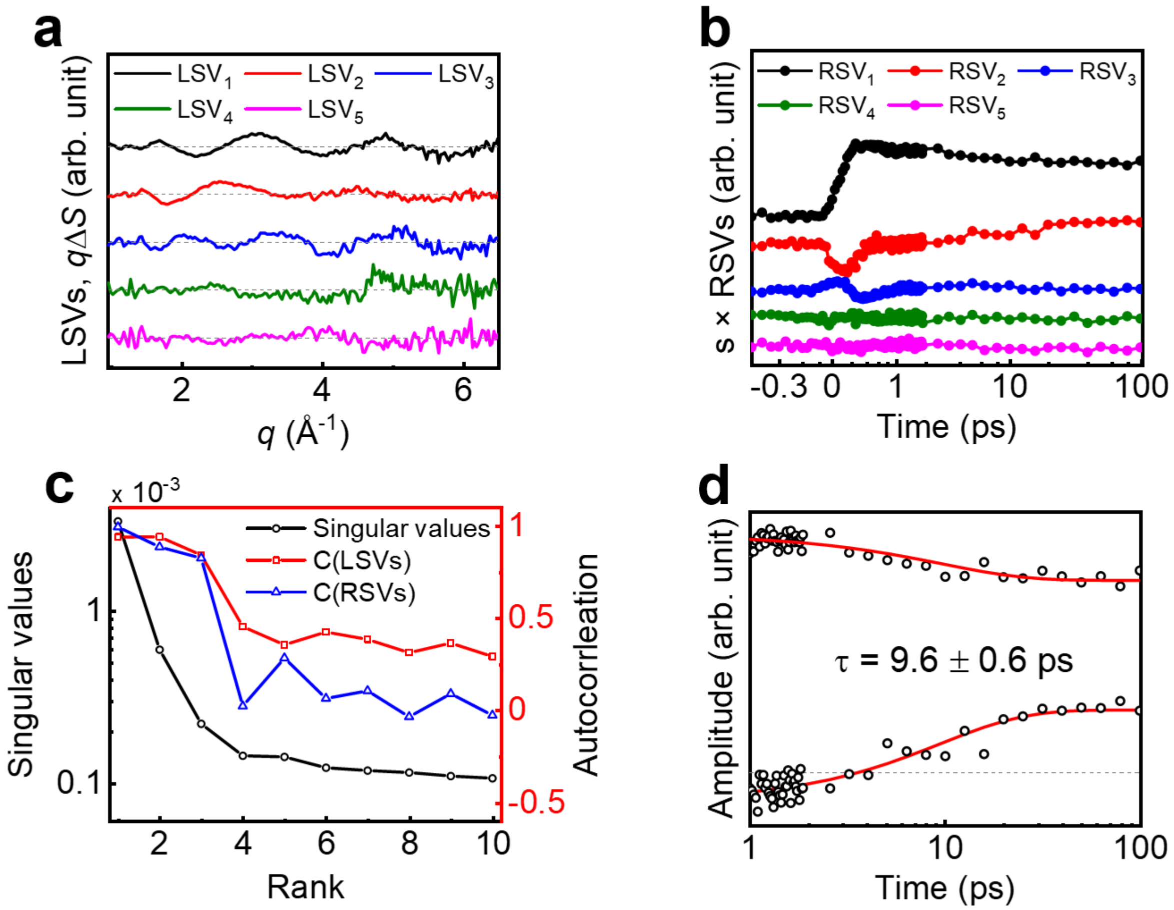

Figure S3. SVD analysis of the isotropic experimental data. (a) The first five left singular vectors (LSVs). (b) The first five right singular vectors (RSVs), weighted by corresponding singular values. The first two RSVs, RSV_1 and RSV_2, contribute to the experimental data across the entire time domain, whereas the third RSV, RSV_3, only contributes during the < 1 ps regime. (c) The first ten singular values, the autocorrelation values of LSVs, and those of RSVs. The first three singular components significantly contribute to the experimental data. (d) The first two RSVs after 1 ps were fitted by single exponential functions with a common time constant, giving $\tau = 9.6 \pm 0.6$ ps.

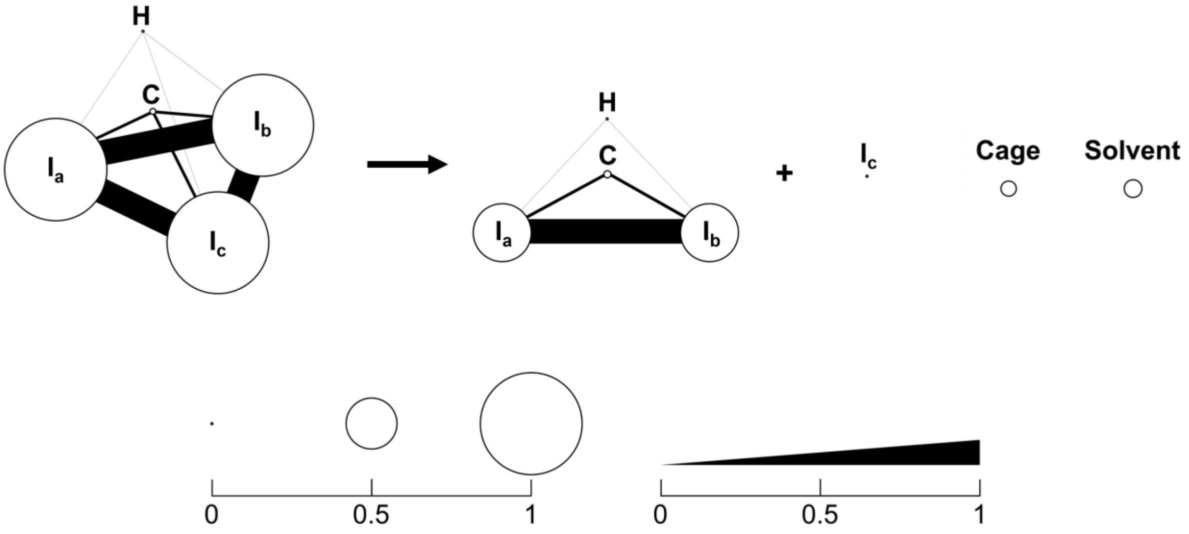

Figure S4. Sensitivity plot for the photoinduced reaction dynamics, CHI_3 → CHI_2• + I• The sensitivity values of the reaction dynamics were calculated via an established method.[11] A nuclear position displacement of 0.1 Å and an internuclear distance change of 0.05 Å were used to estimate the sensitivities of the atomic position and the internuclear distance, respectively. A sensitivity of an atomic position is indicated by a radius of an atom, and a sensitivity of an internuclear distance is indicated by a thickness of a bond. Fig. S11 presents exemplary theoretical scattering curves of the CHI_2 radical with structural variations, highlighting the pronounced differences in sensitivity to changes in internuclear distances.

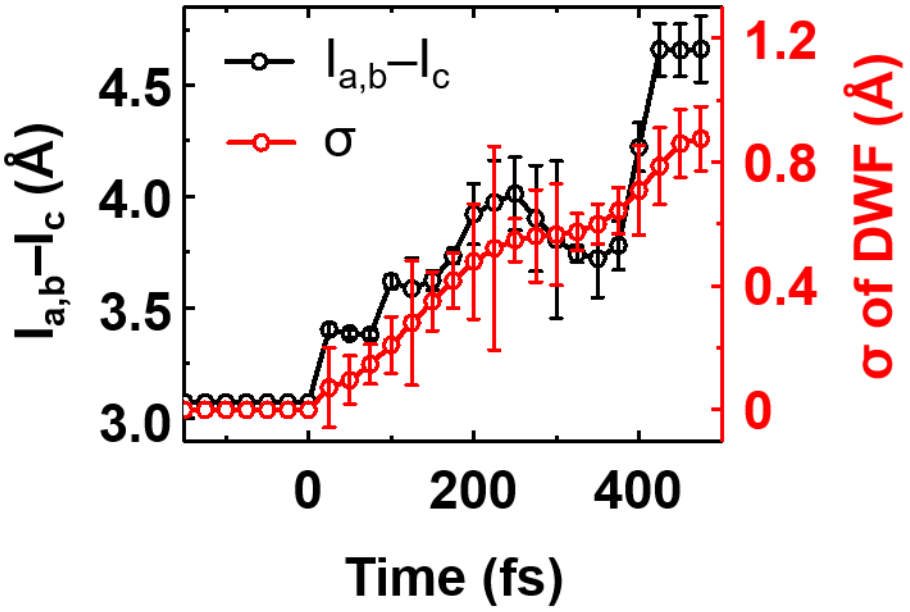

Figure S5. Selected structural parameters from structural analysis at $t \leq 500$ fs. The parameters shown are the $I_{a,b}$–I_c distance and the root-mean-squared displacement (σ) of the Debye-Waller factor (DWF). These parameters are represented by black and red dots, respectively, with corresponding error bars.

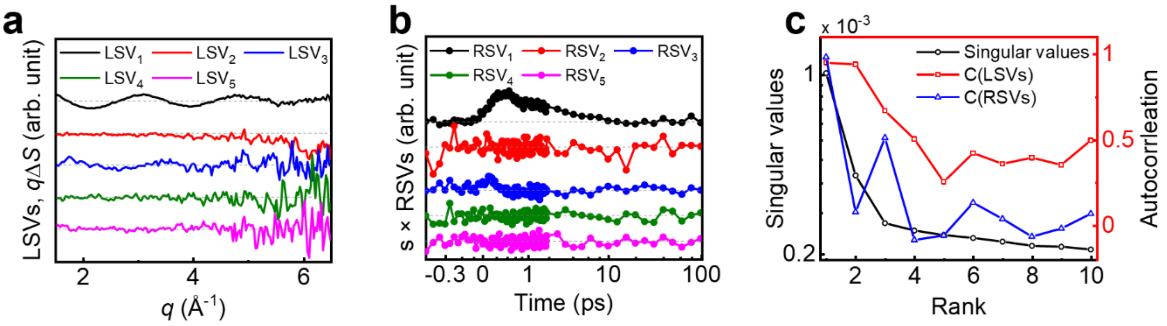

Figure S6. SVD analysis of the anisotropic experimental data. (a) The first five left singular vectors (LSVs). (b) The first five right singular vectors (RSVs), weighted by corresponding singular values. (c) The first ten singular values, the autocorrelation values of LSVs, and those of RSVs. The first singular component significantly contributes to the experimental data. The first RSV after 0.5 ps was fitted by a single exponential function with a time constant, $\tau = 3.0 \pm 0.2$ ps (see Fig. 3d).

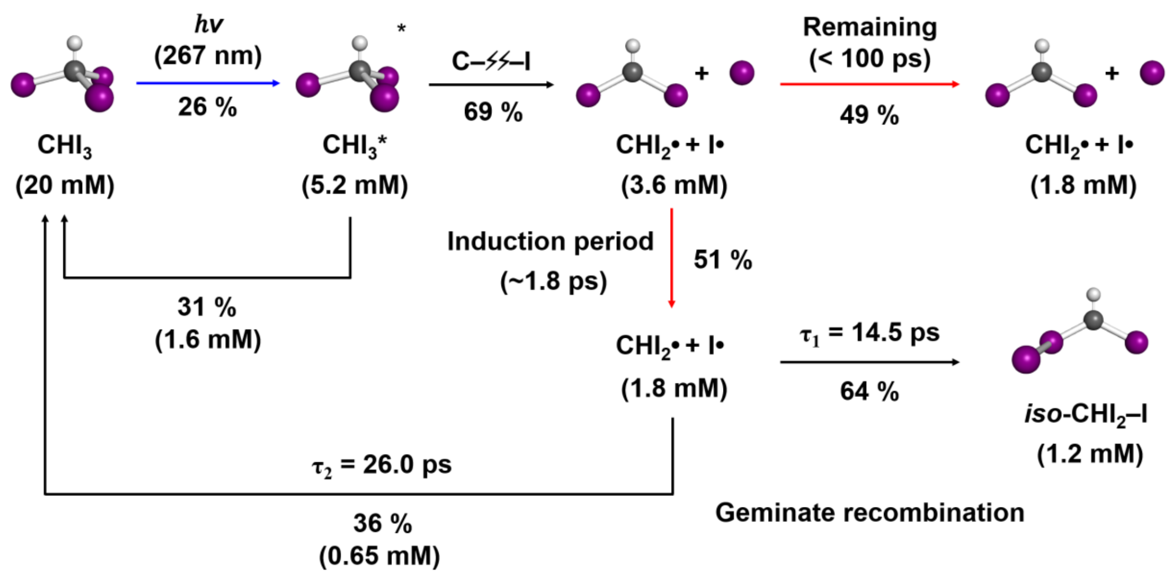

Figure S7. The kinetic framework of CHI_3 photodissociation in cyclohexane with quantitative concentrations of intermediates and pathways determined via fs-TRXL.

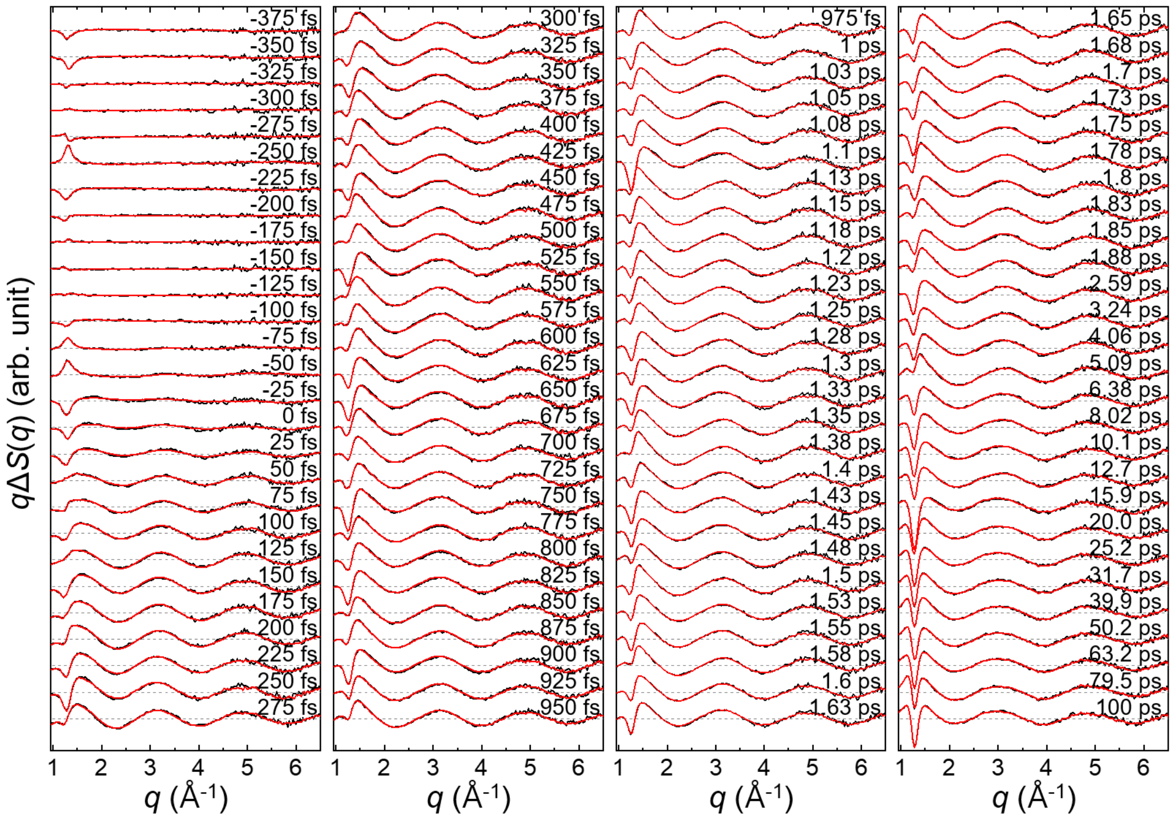

Figure S8. Fit results compared to experimental data. The raw experimental data (black) and the fit results (red) for the time delays from -375 fs to 100 ps. The fit results consist of the artifacts, heating signals, the cage terms, and the Debye curves of intermediate species.

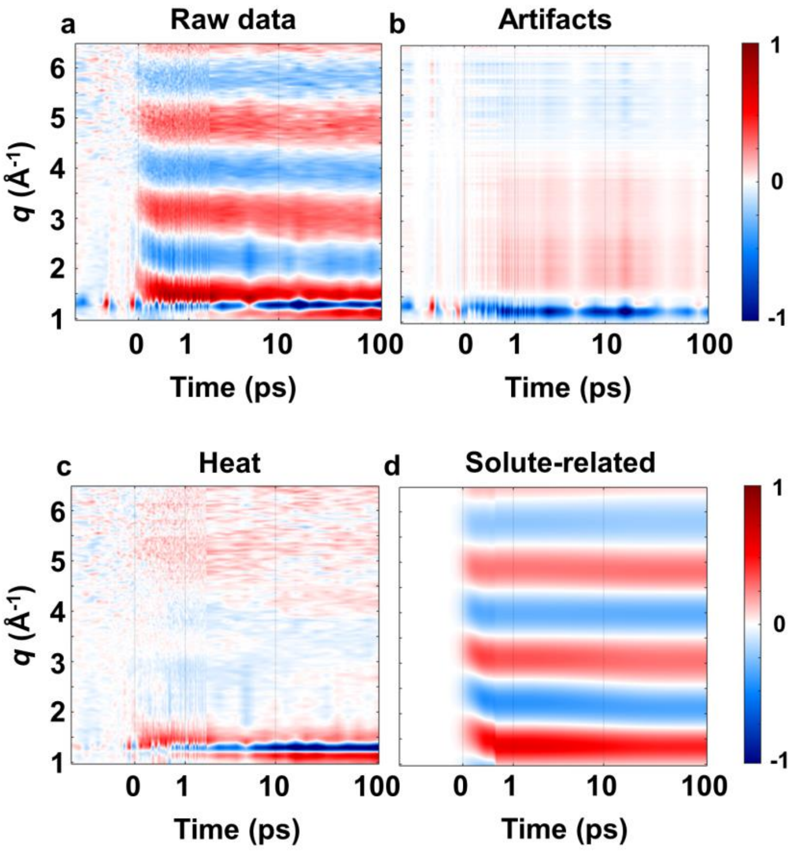

Figure S9. Experimental data and contributions from individual components used in linear combination fitting. (a) The raw experimental data ($q\Delta S_0(q, t)$) presented as a contour map with time (ps) on the *x*-axis and and *q* (Å^{-1}) on the *y*-axis. (b) Contribution from experimental artifacts. (c) Contribution from heating signals. (d) Contribution from solute-related signals.

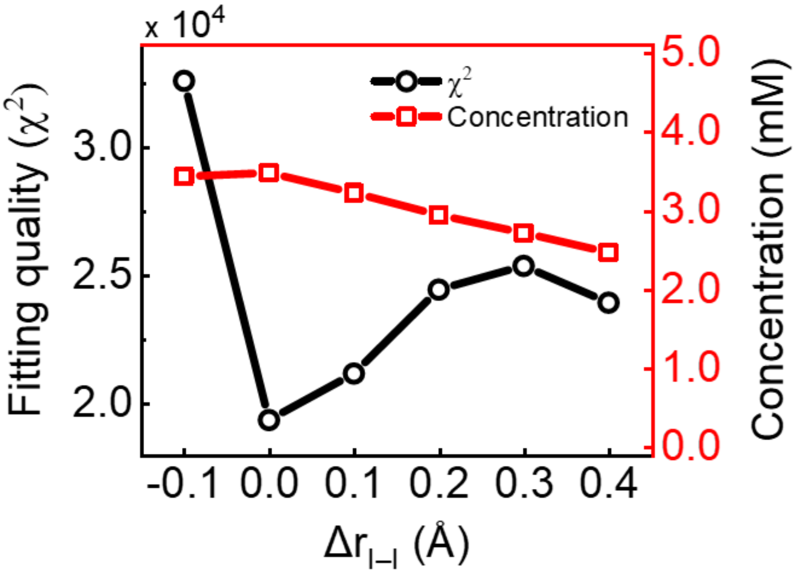

Figure S10. Dual-axis plot showing fitting quality (black, left axis) and excited-state concentration (red, right axis) as functions of the I–I bond distance variation ($\Delta r_{I\text{–}I}$). The significant differences in fitting quality across different structures suggest that adjusting population (excited-state concentration) alone cannot compensate for structural inaccuracies. This indicates the presence of an optimal structure that best explains the experimental data, despite the plausible correlation between species' structures and populations.

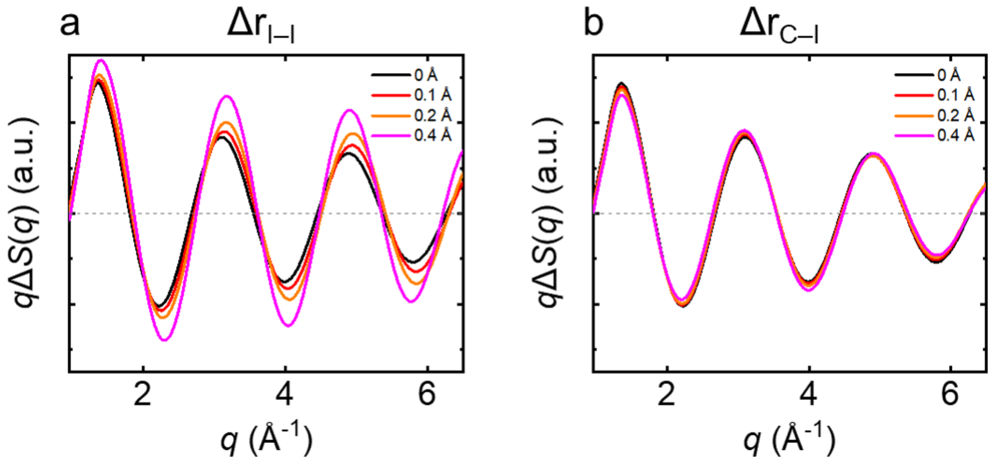

Figure S11. Theoretical scattering curves of CHI_2 radical with structural variations. (a) Scattering curves as the interatomic I–I distance of CHI_2 radical varies from 3.60 to 4.00 Å. (b) Scattering curves as the interatomic C–I distance of CHI_2 radical varies from 2.04 to 2.44 Å.

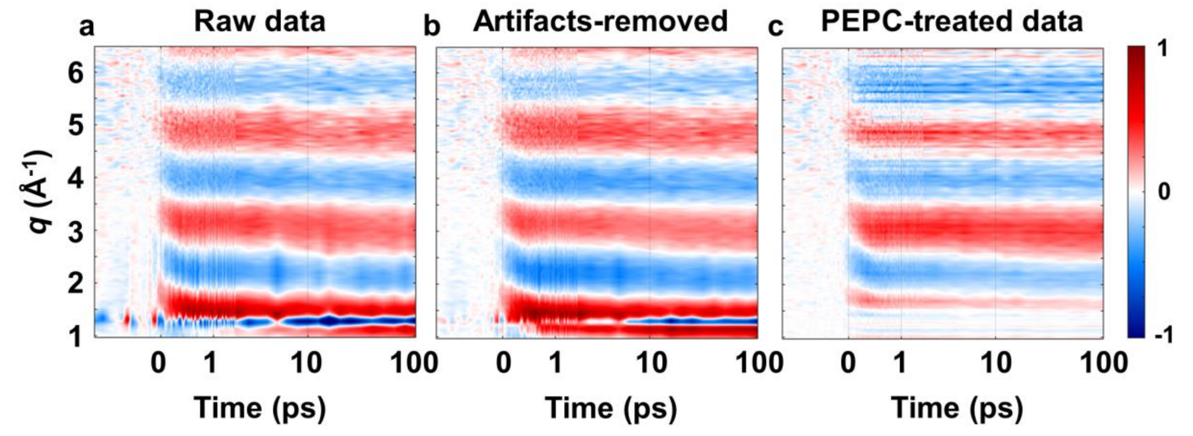

Figure S12. Experimental data and its heat/artifact-filtered versions. (a) The raw experimental data ($q\Delta S_0(q, t)$) presented as a contour map with time (ps) and q (Å^{-1}) as *x*- and *y*-axis, respectively. (b) The data after removing artifacts. (c) The PEPC-treated data ($q\Delta S_0^{\perp}(q, t)$), with both heat and artifacts removed.

Supplementary References

1 I. S. Ko, H.-S. Kang, H. Heo, C. Kim, G. Kim, C.-K. Min, H. Yang, S. Y. Baek, H.-J. Choi, G. Mun, B. R. Park, Y. J. Suh, D. C. Shin, J. Hu, J. Hong, S. Jung, S.-H. Kim, K. Kim, D. Na, S. S. Park, Y. J. Park, Y. G. Jung, S. H. Jeong, H. G. Lee, S. Lee, S. Lee, B. Oh, H. S. Suh, J.-H. Han, M. H. Kim, N.-S. Jung, Y.-C. Kim, M.-S. Lee, B.-H. Lee, C.-W. Sung, I.-S. Mok, J.-M. Yang, Y. W. Parc, W.-W. Lee, C.-S. Lee, H. Shin, J. H. Kim, Y. Kim, J. H. Lee, S.-Y. Park, J. Kim, J. Park, I. Eom, S. Rah, S. Kim, K. H. Nam, J. Park, J. Park, S. Kim, S. Kwon, R. An, S. H. Park, K. S. Kim, H. Hyun, S. N. Kim, S. Kim, C.-J. Yu, B.-S. Kim, T.-H. Kang, K.-W. Kim, S.-H. Kim, H.-S. Lee, H.-S. Lee, K.-H. Park, T.-Y. Koo, D.-E. Kim and K. B. Lee, Construction and commissioning of PAL-XFEL facility, *Appl. Sci.*, 2017, **7**, 479.

2 H.-S. Kang, C.-K. Min, H. Heo, C. Kim, H. Yang, G. Kim, I. Nam, S. Y. Baek, H.-J. Choi, G. Mun, B. R. Park, Y. J. Suh, D. C. Shin, J. Hu, J. Hong, S. Jung, S.-H. Kim, K. Kim, D. Na, S. S. Park, Y. J. Park, J.-H. Han, Y. G. Jung, S. H. Jeong, H. G. Lee, S. Lee, S. Lee, W.-W. Lee, B. Oh, H. S. Suh, Y. W. Parc, S.-J. Park, M. H. Kim, N.-S. Jung, Y.-C. Kim, M.-S. Lee, B.-H. Lee, C.-W. Sung, I.-S. Mok, J.-M. Yang, C.-S. Lee, H. Shin, J. H. Kim, Y. Kim, J. H. Lee, S.-Y. Park, J. Kim, J. Park, I. Eom, S. Rah, S. Kim, K. H. Nam, J. Park, J. Park, S. Kim, S. Kwon, S. H. Park, K. S. Kim, H. Hyun, S. N. Kim, S. Kim, S.-m. Hwang, M. J. Kim, C.-y. Lim, C.-J. Yu, B.-S. Kim, T.-H. Kang, K.-W. Kim, S.-H. Kim, H.-S. Lee, H.-S. Lee, K.-H. Park, T.-Y. Koo, D.-E. Kim and I. S. Ko, Hard X-ray free-electron laser with femtosecond-scale timing jitter, *Nat. Photonics*, 2017, **11**, 708–713.

3 U. Lorenz, K. B. Møller and N. E. Henriksen, On the interpretation of time-resolved anisotropic diffraction patterns, *New J. Phys.*, 2010, **12**, 113022.

4 E. Biasin, T. B. van Driel, G. Levi, M. G. Laursen, A. O. Dohn, A. Moltke, P. Vester, F. B. K. Hansen, K. S. Kjaer, T. Harlang, R. Hartsock, M. Christensen, K. J. Gaffney, N. E. Henriksen, K. B. Møller, K. Haldrup and M. M. Nielsen, Anisotropy enhanced X-ray scattering from solvated transition metal complexes, *J. Synchrotron Radiat.*, 2018, **25**, 306–315.

5 H. Ki, J. Gu, Y. Cha, K. W. Lee and H. Ihee, Projection to extract the perpendicular component (PEPC) method for extracting kinetics from time-resolved data, *Struct. Dyn.*, 2023, **10**, 034103.

6 C. W. Ahn, H. Ki, J. Kim, J. Kim, S. Park, Y. Lee, K. H. Kim, Q. Kong, J. Moon, M. N. Pedersen, M. Wulff and H. Ihee, Direct observation of a transiently formed isomer during iodoform photolysis in solution by time-resolved X-ray liquidography, *J. Phys. Chem. Lett.*, 2018, **9**, 647–653.

7 T. K. Kim, J. H. Lee, M. Wulff, Q. Kong and H. Ihee, Spatiotemporal kinetics in solution studied by time-resolved X-ray liquidography (solution scattering), *ChemPhysChem*, 2009, **10**, 1958–1980.

8 E. H. Choi, J. G. Kim, J. Kim, H. Ki, Y. Lee, S. Lee, K. Yoon, J. Kim, J. Kim and H. Ihee, Filming ultrafast roaming-mediated isomerization of bismuth triiodide in solution, *Nat. Commun.*, 2021, **12**, 4732.

9 K. Refson, Moldy: a portable molecular dynamics simulation program for serial and parallel computers, *Comput. Phys. Commun.*, 2000, **126**, 310–329.

10 W. L. Jorgensen, J. P. Ulmschneider and J. Tirado-Rives, Free energies of hydration from a generalized Born model and an all-atom force field, *J. Phys. Chem. B*, 2004, **108**, 16264–16270.

11 H. Jeong, H. Ki, J. G. Kim, J. Kim, Y. Lee and H. Ihee, Sensitivity of time-resolved diffraction data to changes in internuclear distances and atomic positions, *Bull. Korean Chem. Soc.*, 2022, **43**, 376–390.
